# Poor Accuracy of Blood Pressure Measurement Images Online: Implications for Public Health Education

**DOI:** 10.1161/HYPERTENSIONAHA.125.25064

**Published:** 2025-09-08

**Authors:** Leopold Ndemnge Aminde, Fakir M. Amirul Islam, Victoria E. Cheng, Christina Saad, Yanni Li, Aletta E. Schutte

**Affiliations:** Public Health and Economics Modelling Group, School of Medicine and Dentistry, Griffith University, Gold Coast, Australia (L.N.A., C.S., Y.L.).; School of Health Sciences, Swinburne University of Technology, Hawthorn, Victoria, Australia (F.M.A.I.).; Department of Cardiology, Western Health, Melbourne, Victoria, Australia (V.E.C.).; School of Population Health, University of New South Wales, Sydney, Australia (A.E.S.).; The George Institute for Global Health, Sydney, New South Wales, Australia (A.E.S.).

**Keywords:** blood pressure determination, guideline adherence, health education, internet, photographs, self care

## Abstract

**BACKGROUND:**

Blood pressure (BP) is a common clinical measurement, now increasingly done at home. Media websites often display images of BP measurement to represent clinical medicine, but many images deviate from guidelines, potentially creating misperceptions on how measurement should be performed. We evaluated the accuracy of BP measurement images online according to the 2023 International Consensus on Standardized Clinic BP Measurement.

**METHODS:**

We evaluated the first ≈100 images from each of 11 major stock photo websites. Two independent reviewers assessed each image, resolving disagreements through discussion. Only visible accuracy aspects were scored.

**RESULTS:**

Only 14% of images (N=1106) were accurate on all criteria, ranging from 7% at Flickr and Freepik to 28% at iStock. Photo settings included 63% clinical and 37% home-based; 73% by healthcare providers, 24% by patients, and 3% other settings. Images were penalized for the following guideline deviations: back unsupported (73%), forearm not resting on a surface (55%), manual and not electronic device (52%), feet not flat on the floor (36%), doctor or observer talking/laughing (23%), mid-arm not at heart level (19%), patient talking/laughing (18%), legs crossed (13%), cuff on clothing (12%), and patient not sitting (5%). Accuracy levels differed by setting (clinical 8% versus home 25%, *P*<0.001) and assessors (self 35%, healthcare provider 7%, and other people 13%; *P*<0.001).

**CONCLUSIONS:**

Only 1 in 7 online stock photo images of BP measurement align with clinical guidelines. Media houses, website developers, and the public should be educated on appropriate measurement techniques to ensure accurate BP measurement in the clinic and at home.

**NOVELTY AND RELEVANCE**

**What Is New?**

This study is the first to systematically evaluate online photos depicting blood pressure (BP) measurement on major stock photo websites for their accuracy based on International Consensus Guidelines.

**What Is Relevant?**

BP is commonly measured in clinics and increasingly at home. Inaccurate online depictions can mislead the public, potentially hindering effective BP management.

**Clinical/Pathophysiological Implications?**

Due to the picture-superiority effect, people remember images better than words. With many healthcare professionals not taking BP accurately, and large volumes of erroneous depictions online, there is limited opportunity for patients to ensure that they take their BP accurately at home and that their healthcare providers perform it accurately.

Blood pressure (BP) is highly variable and changes from second-to-second, minute-to-minute, and day-by-day.^1^ Its measurement is perhaps the most common assessment in clinical practice. But if a standard procedure is not followed, the reading will be affected by factors that may lead to erroneous readings of up to +33 or −24 mm Hg,^2^ such as having no rest period before a measurement,^3^ taking only 1 measurement,^4^ not using a device that is validated for accuracy,^5^ using the wrong cuff size,^6^ placing the cuff on top of clothing,^7^ and having the arm resting on the lap or side instead of being supported on a desk.^8^ With clinical guidelines now recommending self-monitoring of BP at home,^9,10^ there is increasing uptake of self-measurement. However, when home BP device owners were asked whether they were taught how to measure BP, <1 in 5 reported having been instructed on several key aspects of self-measurement.^11^

The use of the internet for health information seeking is well documented. A review of 37 studies found that about 80% of users sought health information online.^12^ But it is not known how accurately online images depict BP measurement—even on prestigious and prominent medical platforms. Given that BP measurement is such a key clinical assessment, many websites and news articles often include a stock photo image of a healthcare professional taking BP. Because such images may impact public perception of accurate BP measurement techniques, we evaluated the accuracy of BP measurement images on major stock photo websites according to the criteria of the International Consensus on Standardized Clinical BP Measurement^13^ (Figure 1). We hypothesized that these stock photo images accurately depict BP measurement.

**Figure 1.**
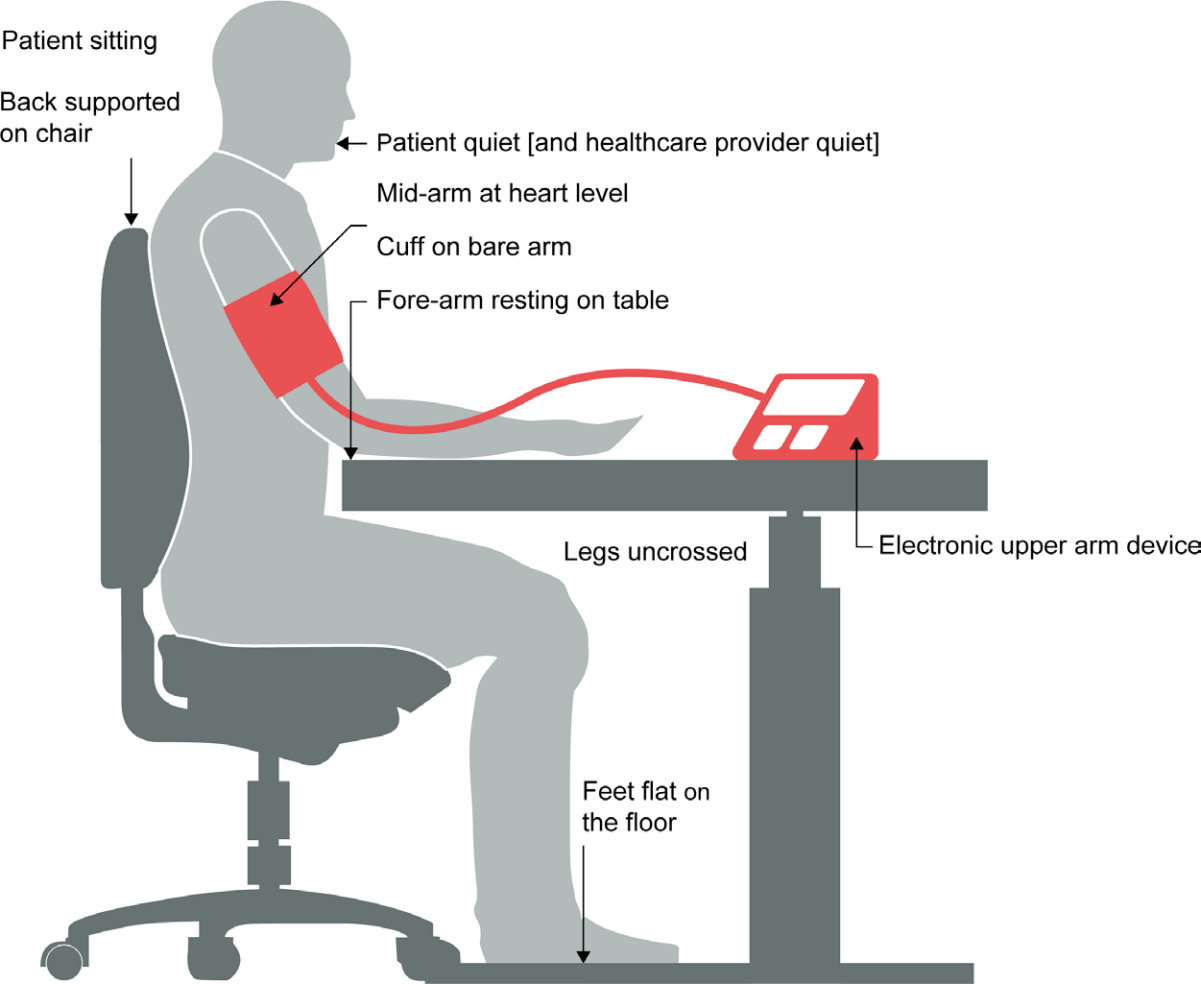
**Visual aspects evaluated on blood pressure measurement accuracy on stock photo images.** Adapted from Unger et al.^14^

## Methods

### Data Availability

Data collected for this study will be made available upon reasonable request by contacting the corresponding author.

### Identification of Stock Photo Sites

**Figure 2.**
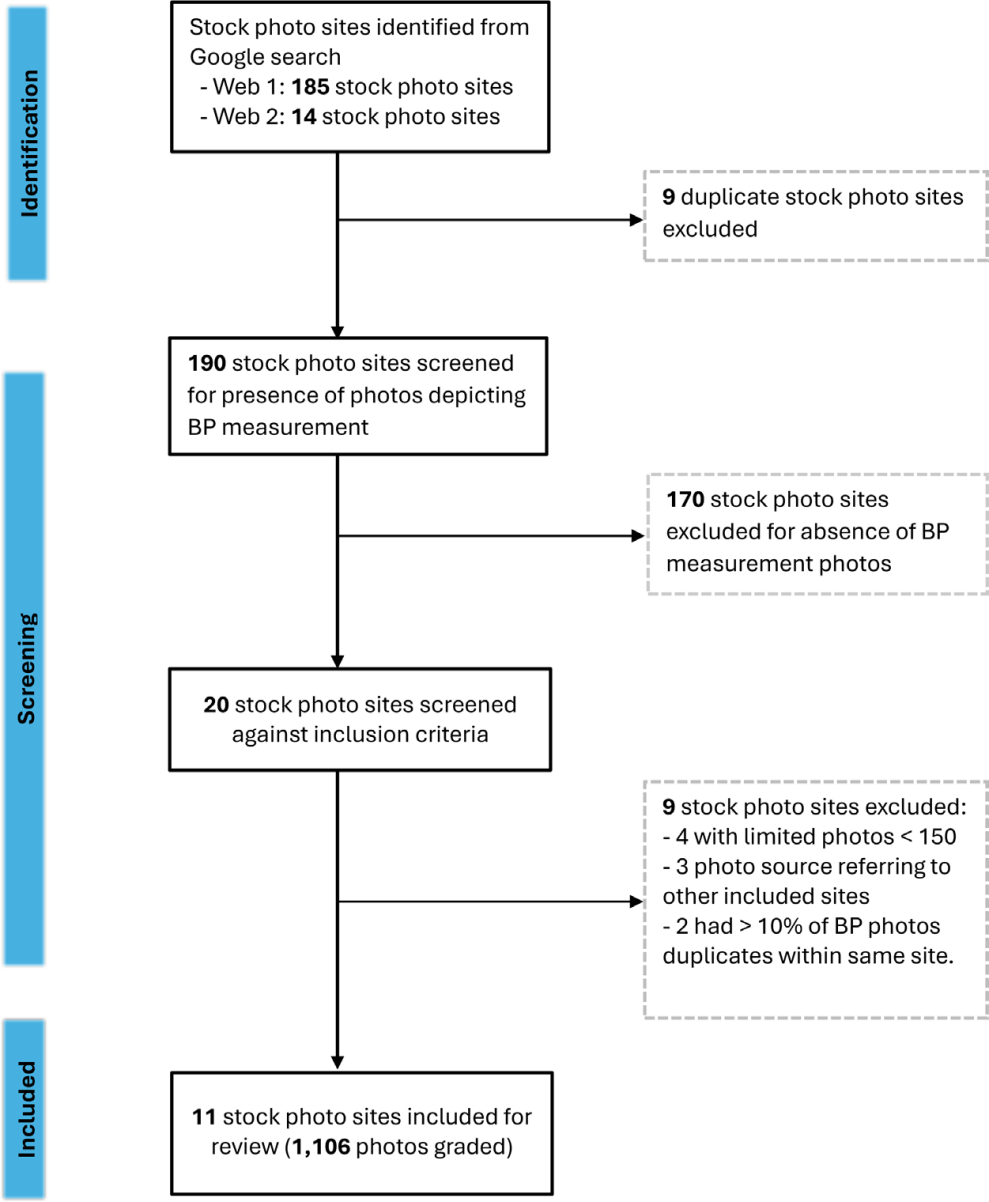
**Flow chart summarizing the selection and inclusion of stock photo sites.** Web 1: https://www.shopify.com/au/blog/17156388-22-awesome-websites-with-stunning-free-stock-images. Web 2: https://traveltractions.com/best-stock-image-websites/. BP indicates blood pressure.

A Google search was conducted on July 22, 2024, to identify a comprehensive list of stock photo sites available online. The following websites (web 1 and web 2) provided extensive lists of stock photo sites available in 2024. Each site was initially screened for potential relevance and inclusion, based on the following criteria: (1) presence of photos depicting BP measurement, (2) contained at least 1000 photos. Figure 2 depicts the flow chart for the identification of the final list of major stock photo sites.

### Search Strategy and Photo Selection

For each included stock photo site, the terms blood pressure check were used to search for photos depicting BP measurement. To be included for further screening, we considered only real photos. We excluded any cartoon or fictional images, artificial intelligence–generated images, or photos without people. The first 100 photos depicting BP measurement in adults from each site were downloaded for further screening. Stock photo sites with >10% duplicate photos were excluded. This was done to limit any potential misestimation of the site-specific and overall photo accuracy levels.

### Screening and Data Extraction

A set of unified criteria was developed (Figure 1), benchmarked against the recommendations for BP measurement specified in the International Consensus on Standardized Clinic BP Measurement,^13^ to guide the screening of photos for their accuracy. All photos were screened independently by 2 reviewers (L.N.A., F.M.A.I., V.E.C., C.S., Y.L.), with each reviewer screening at least 4 stock photo sites. Given that we included 11 stock photo sites (100 photos per site), 1 pair of reviewers screened 5 sites in total. Conflicts were resolved through consensus discussions in 2 stages. First, a pair of reviewers independently screened each photo from the stock photo site. The outcome of this first independent screening process is presented in our agreement statistics. Second, we organized a series of discussion meetings as a whole group to review screening criteria, and then pairs of reviewers for each stock photo site met to discuss photos with conflicting decisions. Here, all photos were once more reviewed for each measurement criterion, and the overall decision on the accuracy (or not) of the photo was reached via consensus.

The following data were extracted: general features: measurement setting (home or clinic/hospital); assessor (self-measured, assisted by a healthcare provider, or another person). The following data on BP measurement deemed assessable from photos were extracted and used as criteria to define photo accuracy: conditions: whether the patient or assessor (where applicable) appear to be talking or laughing; patient position: if sitting or not, whole forearm resting on the table, mid-arm at heart level, back supported on a chair, legs uncrossed, and feet flat on the floor; BP measurement device: using an electronic upper arm device or a manual device; and the BP cuff: whether placed on a bare arm.

### Data and Statistical Analysis

The percentage of agreement and κ statistic were used to evaluate interrater reliability between reviewers. Photo accuracy was determined based on 9 (or 10, when a healthcare provider/another person measured the BP) criteria from the International Consensus on Standardized Clinic BP Measurement.^13^ For each criterion, a score of 1, 0, or 9 was assigned, representing correct, incorrect, or not visually assessable, respectively. A photo was deemed accurate when it scored 1 for each assessable criterion. A score of 0 for any criterion deemed the photo inaccurate, whereas any criterion with a 9 did not penalize the photo. The equation below was used to estimate accuracy per site.


$$\begin{array}{l} \%\;Photo\;\;\;accuracy_{sp}=\;\frac{\;\sum \;Accurate\;\;\;photos_{sp}\;}{\;\sum Total\;\;\;graded\;\;\;photos_{sp}}\;\times \;100 \end{array}$$

where sp=a stock photo site.

Counts and percentages with corresponding 95% CIs were computed to summarize photo accuracy by measurement criterion and by stock photo site. A weighted average was computed to estimate the pooled percentage accuracy across all stock photo sites. χ^2^ tests were used to compare differences in photo accuracy percentage by setting (clinic versus home) and assessor (self-measurement versus healthcare provider/other). Binary logistic regression analysis was conducted to determine factors related to photo accuracy using BP measurement setting and type of assessor as predictors while adjusting for stock photo site. In some photos, BP measurement was done using manual procedures (sphygmomanometer and mercury devices) instead of electronic upper arm devices as specified in the Consensus guideline. We conducted sensitivity analyses reestimating photo accuracy levels and predictors of accuracy, excluding the type of device used from the accuracy criteria. The latter analysis assumed that the manual measurements were done accurately. All analyses were conducted using STATA, version 15.

## Results

### Overview of Included Stock Photos

From 20 potentially relevant stock photo sites identified, 11 were included for screening, namely: 123rf.com, Adobe Stock, Alamy.com, Bigstockphoto, Dreamstime, Flickr, Freepik, Getty Images, iStock, Pikwizard, and Shutterstock. From these, the search returned over 121 000 photos, of which 1106 photos depicting BP measurement in adults were screened (Figure 2; Table 1). The overall unadjusted agreement between reviewers per site was 91.2%. The site-specific κ statistics ranged from 0.39 (95% CI, 0.08–0.69) for Pikwizard to 0.81 (95% CI, 0.63–0.99) for Getty Images, with an overall κ statistic of 0.69 (95% CI, 0.63–0.75), representing moderate agreement (Table 1).

**Table 1.**
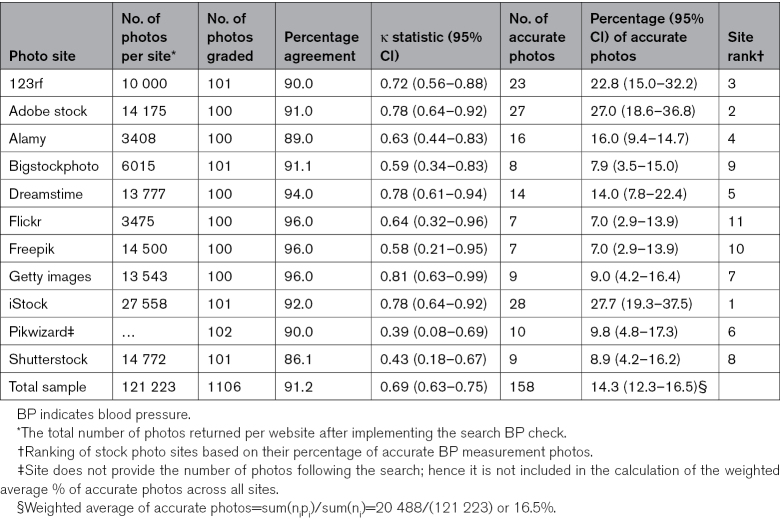
Summary of Interrater Agreement and BP Measurement Photo Accuracy Overall and by Each Stock Photo Site

Overall, 63.2% of the photos were clinic or hospital-based, whereas 36.8% were in the home setting. This varied across stock photo sites, with clinic-based photos ranging from 38.6% in iStock to 86.0% in Flickr. BP measurement was performed by a healthcare provider in 72.8% of photos, whereas 24.5% were done by the patients and 2.7% by other people (Table S1).

### Overall and Site-Specific Photo Accuracy

The proportion of photos across all stock photo sites with accurate BP measurement was 14.3% (95% CI, 12.3–16.5). The site with the highest accuracy was iStock (27.7% [95% CI, 19.3–37.5]), and the lowest were Flickr and Freepik (both, 7.0% [95% CI, 2.9–13.9]). Based on the percentage of accurate photos per site, the top 3 sites were iStock, Adobe Stock, and 123rf, respectively (Table 1). The percentage of photo accuracy was greater in home compared with clinic setting (25.3% versus 7.9%; *P*<0.001) and greater when patients measured their BP compared with when done by a healthcare provider or another person (35.4% versus 7.2% versus 13.3% respectively; *P*<0.001; Table S2). After adjusting for stock photo site, photos depicting BP measurement by the patient themselves or another person had 6-fold higher odds of being accurate compared with those measured by a healthcare provider (odds ratio, 6.97 [95% CI, 4.70–10.33]; Table S3).

**Figure 3.**
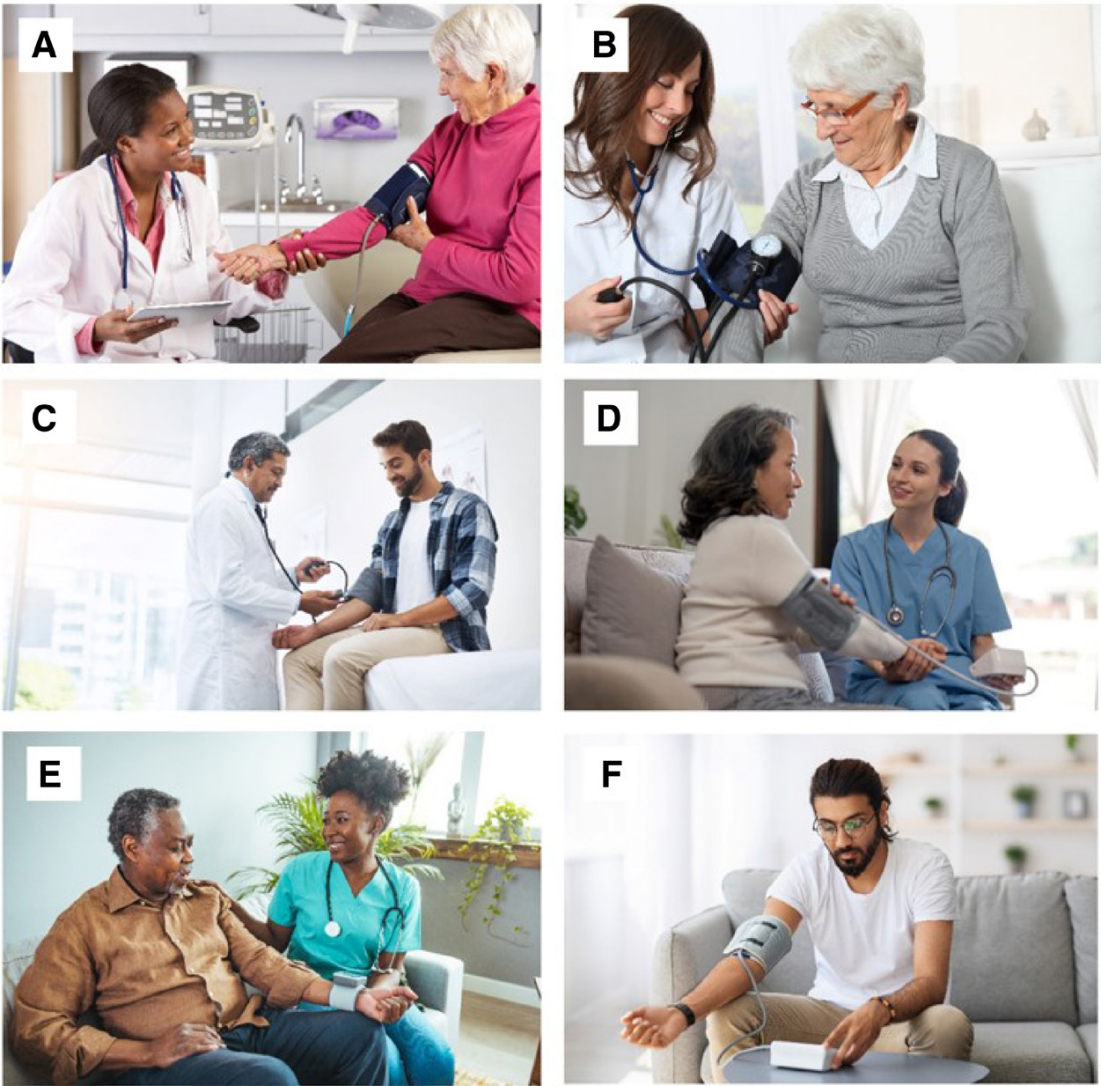
**Examples of stock photo images deviating from clinical guidelines. A**, Clinic blood pressure (BP): back not supported, cuff on clothing, forearm not resting on table, feet not flat on the floor; patient and practitioner likely interacting. **B**, Clinic BP: aneroid device used, cuff on clothing, forearm not resting on table. **C**, Clinic BP: aneroid device used, back not supported, forearm not supported; cuff placement. **D**, Home BP by practitioner: cuff on clothing, forearm not resting on a table, likely speaking and interacting. **E**, Home BP by practitioner: wrist device used and not held at heart level, likely speaking and interacting; **F**, Home BP: back not supported, forearm not supported.

Examples of inaccurate images are shown in Figure 3.

### Sources of Error in BP Measurement

Table 2 shows the different criteria for BP measurement accuracy. Overall, most photos accurately indicated that the patient was sitting during the measurement (95.2%). In 3% (n=33) of all images, the patient was in the supine position.

**Table 2.**
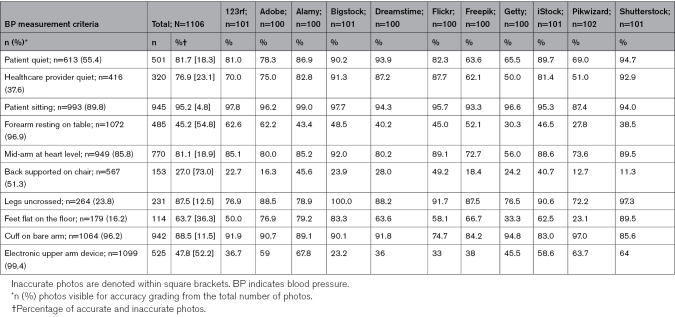
Levels of Accuracy for Each BP Measurement Criterion, by Stock Photo Website

The major deviations from guidelines that contributed to photo inaccuracy included: back not supported on a chair (73.0%), the whole forearm not resting on a table surface (54.8%), manual and not an electronic upper arm device used (52.2%), feet not flat on the floor (36.3%), healthcare provider or the patient talking during procedure (23.1% and 18.3%, respectively), and mid-arm not at heart level (18.9%). In 12.5% of photos, people crossed their legs, and in 11.5% the BP cuff was placed over clothing.

Table S4 shows the association between the assessor and photo setting with BP measurement criteria. The percentages of patient not talking or laughing, patient sitting, having the forearm resting on a table, BP cuff on a bare arm, and use of electronic upper arm device were higher for self-measured BP photos compared with those measured by healthcare providers or other people. The percentage of photos with people’s feet flat on the floor was higher for BP measures by other people and self, compared with healthcare providers. Conversely, the percentage for back supported on a chair was higher in photos with measurement by a healthcare provider or another person compared with self-measurement. For photo setting, the percentage of photos with people accurately having their feet flat on the floor was higher in the home compared with the clinic setting (78.4% versus 46.3%; *P*<0.001). There was no significant difference between settings for all other measurement criteria.

### Sensitivity Analysis

Because some settings may indeed require manual BP measurement, we excluded the type of device used as a criterion for BP measurement accuracy, resulting in an overall accuracy of 26.9% (95% CI, 24.3–29.0). This ranged from 13% (95% CI, 7.1–21.2) in Getty Images to 44.5% (95% CI, 34.6–54.8) in 123rf. Consistent with the primary analysis, adjusted models showed that photos depicting BP measurements done by the patient themselves or another person were more likely to be accurate compared with those measured by a healthcare provider (odds ratio, 2.01 [95% CI, 1.46–2.76]; Tables S3 and S5 through S6).

## Discussion

Our systematic analysis of over 1000 photos of BP measurements across major stock photo websites found only 1 in 7 images aligned with the International Consensus on Standardized Clinic BP Measurement.^13^ The leading errors in BP measurement included the patient’s back being unsupported, their forearm not resting on a surface, not using an electronic BP device, feet not flat on the floor, and the doctor or observer talking or laughing during the procedure. Accuracy levels were higher in the home setting compared with the clinical setting, and higher when BP was self-measured compared with when conducted by healthcare providers.

The value of an accurate BP reading is significantly underestimated. Where blood tests are regarded as set and reliable, the numbers typically produced in practice by an often single BP measurement^4^ simply cannot provide quality data for reliable clinical decision-making. An inaccuracy of 5 mm Hg is estimated to misclassify up to 48 million people in the United States alone.^15^

There may be specific reasons why many clinicians do not follow practice guidelines to measure BP appropriately, such as time constraints or perhaps not understanding the major effect of intraindividual BP variability.^1^ But a global public misconception on several elements required for a reliable BP reading is likely fed by media houses that use stock photo images to support medical content. We were motivated to conduct this review of stock photos, since we observed large volumes of images depicting inaccurate techniques across websites, including prestigious institutions, such as Harvard Health Publishing,^16,17^ Imperial College London,^18^ University College London,^19^ Oxford University,^20^ Stanford University,^21^ and the National Institutes of Health.^22^

It is likely that these images are typically not produced by professionals with an understanding of clinical guidelines and are often purchased by medical journalists with good intentions, but without training to assess images for accurate BP measurement techniques. Furthermore, medical professionals often only review the content of text and not necessarily the images. The media is fully aware of the usefulness of including images with media content due to the picture-superiority effect. This well-known phenomenon refers to people remembering pictures significantly better than they remember corresponding words—and this is intact for children to older people, including people with Alzheimer disease.^23^

Due to the picture-superiority effect, the public health implications of these inaccurate images may be significant. A study including 350 patients performing home BP measurement recently reported that only 1 in 3 received education on how to perform a measurement, but they described it as vague verbal instructions.^24^ In the absence of standardized education, almost all participants reported seeking information online and that they “just worked it out,” “do it the way I’ve seen them do it” by mimicking healthcare professionals and online content.^24^ With many healthcare professionals not taking BP accurately, and with large volumes of erroneous depictions online, there is limited opportunity for patients to take ownership of their health and ensure that they take their BP accurately at home and to ensure that their healthcare providers perform it accurately.

There are many other aspects of performing an accurate clinic or home BP measurement that we were unable to assess on these photos, such as whether a 3 to 5 minute quiet rest period preceded the measurement; whether at least 2 measurements were taken; and whether the correct cuff size was used.^13^ Each of these aspects (and several others) can have a significant effect on the readings,^2^ which is why it is essential that healthcare professionals, patients, and medical journalists undergo appropriate training to ensure that BP measurements are performed and depicted accurately. A basic first step could be for journalists to ensure their images reflect aspects as shown in Figure 1.

We acknowledge that some of the photos included were incomplete. For example, less than a quarter of photos clearly depicted whether the individual had their feet crossed or flat on the floor. It is possible that if these features were visible, they could impact our reported accuracy levels. Although we did not penalize any photo based on features that were not visually assessable, our findings should be interpreted with caution. In addition, we recognize that the stock photographs analyzed in this study were likely neither commissioned nor produced with the 2023 International Consensus on Standardized Clinic BP Measurement in mind, so the errors noted probably do not reflect intentional misrepresentation of proper technique.

If our findings were taken seriously by website developers and media houses, a paradigm shift to improve the quality of BP measurements is possible, as improved quality in images may feed into improved public education and hopefully improved clinical care. Poor clinic BP readings influence treatment decisions, as there seems to be a low confidence in the validity of readings. Treatment inertia, defined as no treatment intensification when BP is above goal, is a well-known consequence of uncertainty in relying on BP readings obtained through poor BP measurement techniques. Of 22 559 primary care visits of patients with uncontrolled hypertension in the United States, only 2138 (10.3%) resulted in treatment intensification.^25^

## Perspectives

With raised BP as the leading cause of preventable deaths in the world,^26^ it is essential to perform accurate measurements in the clinic and at home to ensure clinician confidence to make the required treatment decisions. Our analyses of 1106 photos depicting BP measurement from major stock photo websites found that 6 out of 7 images showed inaccurate measurement techniques. This underscores the pervasiveness of inaccurate visual representations of BP measurement online, which may mislead the public and compromise the correct understanding and practice of BP measurement. Given their key role in accurate visual health communication, we call upon major health organizations, such as the American Heart Association, major media houses, stock photo creators, website developers, medical journalists, and researchers to review their current online images and to ensure that future images depicting BP measurement accurately depict appropriate techniques. Future research should further examine the broader implications of these inaccuracies on public health, self-monitoring practices, and clinical decision-making. Addressing these concerns through systematic investigation and targeted interventions may be beneficial in BP management and in promoting better health outcomes at the population level.

## ARTICLE INFORMATION

### Sources of Funding

A.E. Schutte and L.N. Aminde are funded by Investigator Grants from the National Health and Medical Research Council of Australia (APP2017504 and APP2018082, respectively). L.N. Aminde holds an Honorary Heart Foundation of Australia Fellowship (APP106682).

### Disclosures

A.E. Schutte has received speaker honoraria from Servier, Abbott, Sanofi, AstraZeneca, Medtronic, Omron, and Aktiia; serves on scientific advisory boards for Medtronic, Alnylam/Roche, AstraZeneca, SiSU Health, and Sky Labs; and is cochair of STRIDE BP, an international authority on validated blood pressure devices and measurement techniques (www.stridebp.org). V.E. Cheng has received speaker honoraria from Bristol-Myers Squibb, Boston Scientific, and AstraZeneca. The other authors report no conflicts.

### Supplemental Material

Tables S1–S6

## Supplementary Material


