# Supplementary material for: Poor Accuracy of Blood Pressure Measurement Images Online: Implications for Public Health Education
Source: Hypertension. 2025 Sep 8;82(11):1878–85. doi: 10.1161/HYPERTENSIONAHA.125.25064 (PMC12529977; doi:10.1161/HYPERTENSIONAHA.125.25064)
Supplement: Supplementary file 1 [file hyp-82-1878-s001.docx]

**Appendix**

**Poor Accuracy of Blood Pressure Measurement Images Online: Implications for Public Health Education**

**Leopold N Aminde, MD, PhD^1^, Fakir M Amirul Islam, PhD^2^, Victoria E Cheng, MD^3^, Christina Saad, BMedSci^1^, Yanni Li, PhD^1^, Aletta E Schutte, PhD^4,5^**

^1^ Public Health & Economics Modelling Group, School of Medicine and Dentistry, Griffith University, Gold Coast, Australia

^2^ School of Health Sciences, Swinburne University of Technology, Hawthorn VIC 3122, Australia

^3^ Department of Cardiology, Western Health, Melbourne, Vic, Australia

^4^ School of Population Health, University of New South Wales, Sydney, NSW, Australia

^5^ The George Institute for Global Health, Sydney, NSW, Australia

**Supplementary Table S1: Distribution of photos graded by setting and BP measurement assessor overall and by different sites**

|  | Total N=1106 | | 123rf N=101 | Adobe N=100 | Alamy N=100 | Bigstockphoto N=101 | Dreamstime N=100 | Flickr N=100 | Freepik N=100 | Getty N=100 | iStock N=101 | Pikwizard N=102 | Shutterstock N=101 |
| --- | --- | --- | --- | --- | --- | --- | --- | --- | --- | --- | --- | --- | --- |
|  | **N** | **%** | **%** | **%** | **%** | **%** | **%** | **%** | **%** | **%** | **%** | **%** | **%** |
| *Setting* |  |  |  |  |  |  |  |  |  |  |  |  |  |
| Home | 407 | 36.8 | 19.8 | 45.0 | 17.8 | 56.0 | 32.0 | 14.0 | 31.0 | 44.0 | 61.4 | 43.1 | 40.6 |
| Clinic/hospital | 699 | 63.2 | 80.2 | 55.0 | 82.2 | 44.0 | 68.0 | 86.0 | 69.0 | 56.0 | 38.6 | 56.9 | 59.4 |
| *Assessor* |  |  |  |  |  |  |  |  |  |  |  |  |  |
| Self | 271 | 24.5 | 11.9 | 36.0 | 12.9 | 38.0 | 16.0 | 8.0 | 15.0 | 33.0 | 41.6 | 23.5 | 33.7 |
| Healthcare provider | 805 | 72.8 | 86.1 | 63.0 | 86.1 | 61.0 | 80.0 | 89.0 | 83.0 | 65.0 | 52.5 | 71.6 | 63.4 |
| Another person | 30 | 2.7 | 2.0 | 1.0 | 1.0 | 1.0 | 4.0 | 3.0 | 2.0 | 2.0 | 5.9 | 4.9 | 3.0 |

**Table S2: Percentage of accurate photos by settings and assessors**

|  | No. of photos | Accurate photos | Proportion (95% CI) | P-value |
| --- | --- | --- | --- | --- |
| Total | 1106 | 158 | 14.3 (12.3, 16.5) |  |
| BP measurement setting |  |  |  | <0.001 |
| Home | 407 | 103 | 25.3 (21.1, 29.5) |  |
| Clinic/Hospital | 699 | 55 | 7.9 (5.9, 9.9) |  |
| BP measurement assessor |  |  |  | <0.001 |
| Self | 271 | 96 | 35.4 (29.7, 41.1) |  |
| Health care provider | 805 | 58 | 7.2 (5.4, 9.0) |  |
| Another person | 30 | 4 | 13.3 (1.1, 25.5) |  |

**Table S3: Logistic regression analysis showing predictors of photo accuracy**

|  | Unadjusted models | Adjusted models* |
| --- | --- | --- |
| Primary analysis | **Odds ratio (95% CI)** | **Odds ratio (95% CI)** |
| BP measurement setting |  |  |
| Clinic/Hospital | Ref | Ref |
| Home | 3.97 (2.78 – 5.66) | 4.06 (2.76 – 5.97) |
| BP measurement assessor |  |  |
| Healthcare provider | Ref | Ref |
| Self or another person | 6.41 (4.47 – 9.17) | 6.97 (4.70 – 10.33) |
| Sensitivity analysis ^#^ |  |  |
| BP measurement setting |  |  |
| Clinic/Hospital | Ref | Ref |
| Home | 1.20 (0.91 – 1.58) | 1.30 (0.96 – 1.76) |
| BP measurement assessor |  |  |
| Healthcare provider | Ref | Ref |
| Self or another person | 1.69 (1.27 – 2.26) | 2.01 (1.46 – 2.76) |

Note: due to small counts, the assessor category ‘another person’ was merged with the self-measure category.

* Odds ratios and 95% confidence intervals are adjusted for stock photo site.

^#^ Photo accuracy levels were re-estimated after excluding the type of BP measurement device as a criterion for accuracy.

**Table S4: Blood pressure measurement accuracy criteria by photo setting and type of assessor**

|  | Setting | | | Assessors | | | |  |
| --- | --- | --- | --- | --- | --- | --- | --- | --- |
|  | **Home**  **N=407** | **Clinic**  **N=699** |  | **Self**  **N=271** | **Healthcare provider**  **N=805** | **Other**  **N=30** |  |  |
|  | **%** | **%** | **p-value** | **%** | **%** | **%** | **p-value** |  |
| Patient quiet | 84.1 | 80.1 | 0.21 | 92.3 | 79.2 | 66.7 | <0.001 |  |
| Healthcare provider quiet | 73.3 | 78.1 | 0.32 | 100 | 77.4 | 61.9 | 0.12 |  |
| Patient sitting | 95.8 | 94.8 | 0.48 | 98.2 | 94.2 | 96.7 | 0.04 |  |
| Forearm resting on table | 44.6 | 45.6 | 0.75 | 53.0 | 42.7 | 40.0 | 0.01 |  |
| Mid-arm at heart level | 79.6 | 82.0 | 0.36 | 79.9 | 81.7 | 75.9 | 0.64 |  |
| Back supported on a chair | 26.2 | 27.5 | 0.72 | 21.4 | 27.8 | 50.0 | 0.02 |  |
| Legs uncrossed | 86.4 | 88.7 | 0.57 | 84.2 | 88.9 | 100.0 | 0.33 |  |
| Feet flat on the floor | 78.4 | 46.3 | <0.001 | 80.0 | 53.1 | 100.0 | <0.001 |  |
| Cuff on bare arm | 90.6 | 87.4 | 0.11 | 94.9 | 86.9 | 76.7 | <0.001 |  |
| Electronic upper arm device | 80.9 | 28.4 | <0.001 | 94.8 | 30.7 | 79.3 | <0.001 |  |

**Table S5: Sensitivity analysis for photo accuracy after excluding the type of BP device used as a criterion for measurement accuracy**

| Stock photo site | Total photos^a^ | Photos graded | Accurate photos | | Inaccurate photos | | | Site rank^c^ |
| --- | --- | --- | --- | --- | --- | --- | --- | --- |
|  |  |  | Count | Percentage (95% CI) | Count | | Percentage (95% CI) |  |
|  |  |  |  |  |  |  | |  |
| 123rf | 10,000 | 101 | 45 | 44.5 (34.6 – 54.8) | 56 | 55.4 (45.2 – 65.3) | | 1 |
| Adobe stock | 14,175 | 100 | 40 | 40.0 (30.3 – 50.3) | 60 | 60.0 (49.7 – 69.7) | | 2 |
| Alamy | 3,408 | 100 | 28 | 28.0 (19.5 – 37.9) | 72 | 72.0 (62.1 – 80.5) | | 6 |
| Bigstockphoto | 6,015 | 101 | 34 | 33.7 (24.6 – 43.8) | 67 | 66.3 (56.2 – 75.4) | | 5 |
| Dreamstime | 13,777 | 100 | 24 | 24.0 (16.0 – 33.6) | 76 | 76.0 (66.4 – 83.9) | | 7 |
| Flickr | 3,475 | 100 | 18 | 18.0 (11.0 – 26.9) | 82 | 82.0 (73.1 – 88.9) | | 8 |
| Freepik | 14,500 | 100 | 37 | 37.0 (27.6 – 47.2) | 63 | 63.0 (52.8 – 72.4) | | 3 |
| Getty Images | 13,543 | 100 | 13 | 13.0 (7.1 – 21.2) | 87 | 87.0 (78.8 – 92.8) | | 10 |
| iStock | 27,558 | 101 | 36 | 35.6 (26.4 – 45.8) | 65 | 64.4 (54.2 – 73.6) | | 4 |
| Pikwizard^d^ | - | 102 | 14 | 13.7 (7.7 – 21.9) | 88 | 86.3 (78.0 – 92.3) | | 9 |
| Shutterstock | 14,772 | 101 | 9 | 8.9 (4.1 – 16.2) | 92 | 91.1 (83.8 – 95.8) | | 11 |
|  |  |  |  |  |  |  | |  |
| Total sample | 121,223 | 1106 | 298 | 26.9 (24.3 – 29.0) | 808 | 73.1 (70.3 – 75.7) | | - |
| Weighted average % |  |  |  | 29.1 (28.8 – 29.4) |  |  | |  |

^a^ The total number of photos returned per website after implementing the search ‘blood pressure check’.

^b^ Refers to the projected number of accurate BP measurement photos estimated as the product of the percentage accuracy and total number of photos per site.

^c^ Ranking of stock photo sites based on their percentage of accurate BP measurement photos.

^d^ Site does not provide the number of photos following the search; hence it is not included in the calculation of the weighted average % of accurate photos across all sites.

**Table S6: Sensitivity analysis of the percentage of accurate BP measurement photos by setting and assessor after excluding type of BP device as a criterion for measurement accuracy**

|  |  | Accurate | | Inaccurate | |  |
| --- | --- | --- | --- | --- | --- | --- |
|  | **N** | **n** | **% (95% CI)** | **n** | **% (95% CI)** |  |
| Total | 1106 | 298 | 26.9 (24.3 – 29.7) | 808 | 73.1 (70.3 – 75.7) |  |
| Setting |  |  |  |  |  | p = 0.189 |
| Home | 407 | 119 | 29.2 (24.9 – 33.9) | 288 | 70.8 (66.1 – 75.1) |  |
| Clinic | 699 | 179 | 25.6 (22.4 – 29.0) | 520 | 74.4 (70.9 – 77.6) |  |
| Assessor |  |  |  |  |  | p <0.001 |
| Self | 271 | 101 | 37.3 (31.5 – 43.3) | 170 | 62.7 (56.7 – 68.5) |  |
| Healthcare provider | 805 | 193 | 23.9 (21.1 – 27.1) | 612 | 76.0 (72.9 – 78.9) |  |
| Another person | 30 | 4 | 13.3 (3.7 – 30.7) | 26 | 86.7 (69.3 – 96.2) |  |
